# Different Cortical Mechanisms for Spatial vs. Feature-Based Attentional Selection in Visual Working Memory

**DOI:** 10.3389/fnhum.2016.00415

**Published:** 2016-08-17

**Authors:** Anna Heuer, Anna Schubö, J. D. Crawford

**Affiliations:** ^1^Experimental and Biological Psychology, Philipps-University MarburgMarburg, Germany; ^2^Centre for Vision Research, York UniversityToronto, ON, Canada; ^3^Canadian Action and Perception Network, York UniversityToronto, ON, Canada; ^4^Departments of Psychology, Biology, and Kinesiology and Health Sciences, York UniversityToronto, ON, Canada

**Keywords:** working memory, attention, transcranial magnetic stimulation, spatial attention, feature-based attention, retrocue

## Abstract

The limited capacity of visual working memory (VWM) necessitates attentional mechanisms that selectively update and maintain only the most task-relevant content. Psychophysical experiments have shown that the retroactive selection of memory content can be based on visual properties such as location or shape, but the neural basis for such differential selection is unknown. For example, it is not known if there are different cortical modules specialized for spatial vs. feature-based mnemonic attention, in the same way that has been demonstrated for attention to perceptual input. Here, we used transcranial magnetic stimulation (TMS) to identify areas in human parietal and occipital cortex involved in the selection of objects from memory based on cues to their location (spatial information) or their shape (featural information). We found that TMS over the supramarginal gyrus (SMG) selectively facilitated spatial selection, whereas TMS over the lateral occipital cortex (LO) selectively enhanced feature-based selection for remembered objects in the contralateral visual field. Thus, different cortical regions are responsible for spatial vs. feature-based selection of working memory representations. Since the same regions are involved in terms of attention to external events, these new findings indicate overlapping mechanisms for attentional control over perceptual input and mnemonic representations.

## Introduction

Visual working memory (VWM) allows us to maintain and manipulate visual information over short periods of time for various cognitive and motor tasks. However, this critical function has a highly limited capacity (Luck and Vogel, 1997; Zhang and Luck, 2008). As a result of this limitation, it is important for the brain to continuously and selectively update the contents held in VWM, thereby improving memory for some objects at the expense of others (e.g., Kuo et al., 2012; Zokaei et al., 2014). Further, in order to achieve the flexibility required for different tasks, this selection process must operate in different qualitative domains, such as spatial vs. featural information (Pertzov et al., 2013; Li and Saiki, 2014; Heuer and Schubö, 2016a). However, the neural mechanisms used to deploy such differential selection are unknown at this time.

By comparison, much more is known about selective attention in perception. In particular, it has been shown that spatial and feature-based perceptual attention have different behavioral consequences and different neural mechanisms (e.g., Maunsell and Treue, 2006; Schenkluhn et al., 2008; Greenberg et al., 2010; Carrasco, 2011). Perceptual and mnemonic attentional selection have been shown to exhibit many commonalities, but there are also notable differences in terms of behavioral signatures (e.g., Tanoue and Berryhill, 2012) and in terms of cortical mechanisms (Nobre et al., 2004; Nee and Jonides, 2009; Tamber-Rosenau et al., 2011). Therefore, one cannot assume that mnemonic and perceptual attentions share the same feature-specific cortical mechanisms.

In the present study, we used structural magnetic resonance imaging (MRI)-guided, on-line repetitive transcranial magnetic stimulation (rTMS) to test whether spatial and feature-based attention to remembered visual objects can be dissociated based on the site of cortical stimulation. On-line transcranial magnetic stimulation (TMS) can transiently disrupt activity in a localized brain region, thereby establishing a causal, spatiotemporal link between this region and cognitive functions engaged at that point in the task (e.g., Hallett, 2000; Bolognini and Ro, 2010). In a change-detection task, participants were required to remember the colors of three differently shaped items, and then report whether there was a color change for one of the items. The items were presented either in the left or in the right visual hemifield to allow for an investigation of a potential lateralization with respect to the stimulated right hemisphere. A lateralization of attentional selection in VWM has previously been observed in electrophysiological studies (Griffin and Nobre, 2003; Poch et al., 2014; Myers et al., 2015). The right hemisphere was chosen for stimulation, because the attentional network has often been shown to be right-hemisphere dominant (e.g., Corbetta and Shulman, 2002; Thiebaut de Schotten et al., 2011; Chang et al., 2013). During the retention interval, a so-called “retrocue” was presented, that is, a cue indicating specific previously-presented items as more behaviorally relevant than others. This retrocue indicated the upcoming test item either by its location (spatial attention) or by its shape (feature-based attention). Based on previous studies (Pertzov et al., 2013; Li and Saiki, 2014; Heuer and Schubö, 2016a), we expected a general improvement in performance in cued compared to that in neutral control trials for both feature-based and spatial retrocues. We then selectively targeted the cortical mechanisms for spatial vs. feature-based attentional selection by delivering a short train of three TMS pulses to the right supramarginal gyrus (SMG) or the right lateral occipital cortex (LO) during presentation of the retrocue. These areas were chosen based on what is known about their roles in perceptual attention. Whereas parietal SMG has been implicated in the control of spatial attention (Chambers et al., 2004; Schenkluhn et al., 2008), extrastriate visual cortex is involved in feature-based attention (Corbetta et al., 1991; Murray and Wojciulik, 2004; Schoenfeld et al., 2007), with LO playing a specific role in the representation of object shape (Kourtzi and Kanwisher, 2000; Grill-Spector et al., 2001; Kim et al., 2011). If these areas play similar roles in the differential selection of mnemonic representations, stimulation of SMG vs. LO during the cue presentation should produce differential effects on attentional selection based on location vs. shape, thus dissociating spatial and feature-based attention in VWM at the cortical level.

## Materials and Methods

### Participants

Eleven volunteers (7 females; mean age: 27 years, *SD* = 6 years; two left-handed) participated in the experiment. All participants were in good health, had normal or corrected-to-normal visual acuity and color vision and according to self-report, no known contraindications to TMS. Participants provided informed written consent before the experiment but were otherwise naive to the purpose of the study. The procedures were approved by the York University Human Participants Review Subcommittee and were in accordance with the Declaration of Helsinki.

### Apparatus

Participants were seated in a dimly-lit room, facing a CRT monitor (19″, frame rate 85 Hz) placed at a distance of approximately 100 cm from their eyes. During the experiment, their head was fixed in an upright position centrally to the monitor by individual dental impressions (bite bars). Participants responded by pressing two buttons on a keyboard placed on a table in front of them with the index and middle fingers of their right hand. Stimulus presentation and response collection were controlled by a Windows PC using E-Prime 2.0 software (Psychology Software Tools, Inc.).

### Stimuli and Task

**Figure 1:**
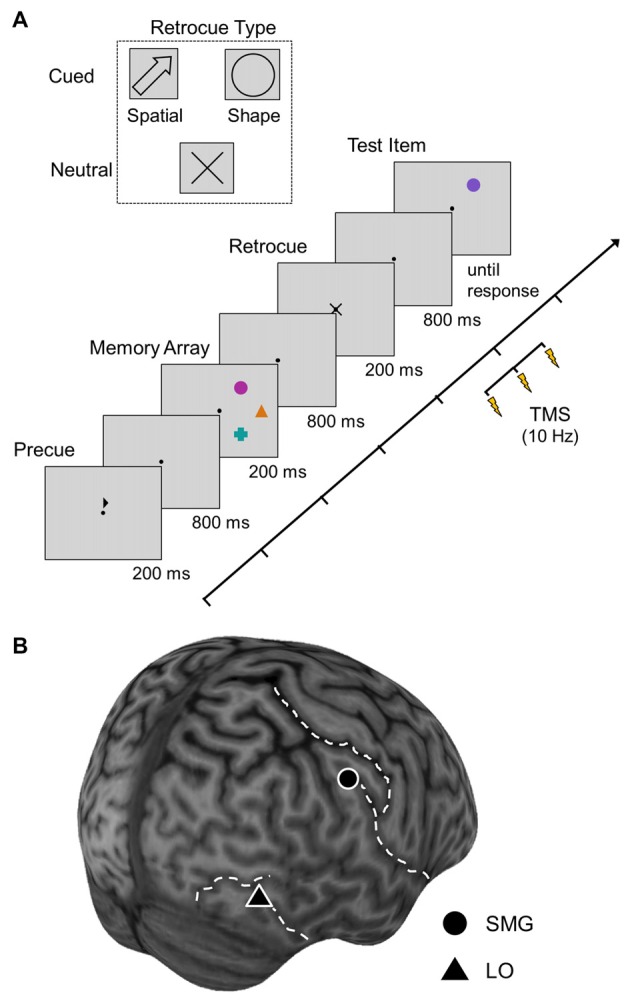
**Task and transcranial magnetic stimulation (TMS) protocol. (A)** A trial for the right hemifield. Participants memorized colors of the items in the memory array, and indicated whether the test item had the same color as the item previously presented at that location. In cued trials, the retrocue indicated the test item by its location or shape. In neutral trials, the retrocue was uninformative. In TMS conditions, a train of three pulses was applied during retrocue presentation. The first pulse was delivered 100 ms after retrocue onset. **(B)** Location of TMS sites supramarginal gyrus (SMG) and lateral occipital cortex (LO) in the right hemisphere of one participant. Dashed lines indicate the sulci that were used to identify the sites.

All stimuli were presented against a gray background and participants were instructed to remain focused on a central dot (0.8° of visual angle) throughout the experimental trials. Our visual stimuli and task are most easily described in terms of the temporal sequence of steps illustrated in Figure 1A.

Step 1: A trial started with the presentation of a precue (an arrowhead subtending 0.94° × 0.50°) above the fixation dot for 200 ms, which pointed towards the left or right, thereby indicating the relevant visual hemifield for that trial. This precue allowed participants to selectively allocate attention to the correct hemifield, facilitating the upcoming encoding process and reducing the likelihood of eye movements toward transiently presented memory items.

Steps 2 and 3: After an interval of 800 ms, the memory array was presented, which consisted of three memory items in the relevant hemifield. Participants were instructed to memorize the colors of these items. Memory items subtended an area of 1.10° of visual angle and were arranged on an imaginary circle with a radius of 4.96° and with a distance of 3.58° between items. The colors of the memory items were randomly chosen from a set of seven colors (magenta, violet, blue, turquoise, green, orange, and red) with the restriction that no two memory items could be of the same color. The number of memory items was close to the capacity limit of VWM (e.g., Luck and Vogel, 1997), and the colors were adjusted so that the baseline performance was within the optimal zone of difficulty for TMS effects on working memory (see Prime et al., 2008, 2010). The shapes of the memory items were chosen from a set of four shapes (circle, cross, square, and triangle). On a given trial, all memory items were of different shapes. All 24 possible combinations of locations and shapes were presented equally often and in a randomized order.

Steps 4 and 5: After 800 ms, the retrocue (0.83°) was presented for 200 ms (see Figure 1A, upper panel for details of the retrocue stimulus appearance). In cued trials, the retrocue indicated one of the memorized items by either its location (spatial retrocue) or by its shape (shape retrocue). Participants were informed that this was the item that would be tested at the end of the trial. In neutral trials, a non-informative retrocue was presented (an “X”).

Steps 6 and 7: After another interval of 800 ms, the test item was presented at one of the memory item locations, and participants had to indicate whether this item was of the same or of a different color as the memory item that had previously been presented at that location. In cued trials, the test item was presented at the location of the cued item. All locations were equally likely to be tested, but chosen in a randomized order. The color of the test item was either identical to the color of the memory item that had previously been presented at that location or a different, spectrally neighboring color. The shape of the test item was always that of the memory item that had previously been presented at the respective location. The test item was present until response, but a quick decision was encouraged. Participants responded by pressing a button with their right index or middle finger, and the response assignment was balanced across participants.

In no-TMS trials, the inter-trial interval (ITI) was 1 s. For safety reasons, the ITI was increased to 10 s in TMS blocks. A separate control experiment (see “ITI Control Experiment” in Materials and Methods Section and “ITI Control Experiment” in Results Section) was conducted to investigate the effects of these different ITI durations.

### Design

The experiment consisted of 864 trials. There were 288 trials for each TMS condition (noTMS, LO, and SMG) with 144 trials for each retrocue type (spatial and shape), half of which were cued and the other half neutral. Retrocue type was varied block-wise and changed with every three blocks of 24 trials each. A block design was chosen, because this has previously been shown to yield significant benefits for different types of retrocues (Li and Saiki, 2014; Heuer and Schubö, 2016a), whereas a study using a trial-by-trial change failed to observe benefits for retrocue types that were not directly spatial (Berryhill et al., 2012). The order in which the retrocue types were presented was balanced across participants. Cued and neutral trials were randomly interleaved within these blocks.

Testing took place in four sessions in consecutive weeks. Each session started with three noTMS blocks, followed by six blocks with TMS: in the first two sessions one TMS site was stimulated and in the last two the other TMS site. The order in which the two TMS sites were stimulated was balanced across participants. We did not use separate TMS sites or sham TMS as controls, because the design aimed at a double dissociation: the two sites provided controls for each other and for any non-specific effects of TMS (e.g., the clicking sound of the TMS coil), which would affect either both or none of the stimulation sites. Similar designs have been successfully used in other TMS studies (e.g., Pelgrims et al., 2009; Pitcher et al., 2009; Malik et al., 2015). Prior to the first session, every participant completed a short training session on a separate day.

### ITI Control Experiment

Sixteen volunteers (14 females; mean age: 21 years, *SD* = 3 years; one left-handed) participated in the control experiment. None of them had also participated in the main experiment. Stimuli, task and design were the same as in the main experiment, except for the following. The experiment consisted of 288 trials. For one half of the experiment, the ITI was long (10 s, as in the TMS blocks in the main experiment), and for the other half of the experiment, the ITI was short (1 s, as in the noTMS blocks in the main experiment). The order of long and short ITIs was balanced across participants. The *d′* scores were calculated separately for long and short ITIs, and for cued and neutral trials.

### Localization of Brain Sites and TMS Protocol

To localize the stimulation sites and monitor the TMS coil position, a frameless stereotaxic neuronavigation system (Brainsight, Rogue Research, Montréal, QC, Canada) was used. Three-dimensional structural T1-weighted MRIs were obtained for all participants prior to the behavioral sessions. The two stimulation sites in the right hemisphere were identified individually for each participant according to anatomical criteria and based on previous studies (Chambers et al., 2007; Large et al., 2007; Cohen et al., 2009). SMG was defined as the region adjacent to the dorsolateral projection of the lateral sulcus, posterior to the post-central sulcus and anterior to the superior temporal sulcus (average Talairach coordinates: 49, −33, 37; average MNI coordinates: 54, −31, 39). LO was near the junction of the inferior temporal sulcus and the lateral occipital sulcus (average Talairach coordinates: 37, −70, −2; average MNI coordinates: 40, −73, −1). Figure 1B shows the stimulation sites in the right hemisphere of one participant.

In each trial of the TMS blocks, a repetitive pulse train consisting of three pulses with a frequency of 10 Hz was delivered 100 ms after cue onset. Stimulation intensity was fixed to 60% of the stimulator output. These stimulation parameters were chosen based on previous studies (Chambers et al., 2007; Schenkluhn et al., 2008; Pitcher et al., 2009; Mullin and Steeves, 2011). The delay of 100 ms between retrocue presentation, and the following timing of the three pulses ensured that the stimulation did not affect perceptual processing of the retrocue, but effectively covered the temporal range of its attentional processing (see also Souza and Oberauer, 2016). TMS was administered using a Magstim Rapid 2 system and a 70 mm figure-of-eight coil that was held tangentially to the scalp surface.

### Data Analysis

Trials with excessively long reaction times (>2.5 SD from mean RT calculated individually for each participant) were excluded from further analysis (on average, 3% of all trials). The dependent variable for all analyses was the sensitivity of change detection (*d′*). The *d′* scores were calculated as *d′* = z(hit rate) − z(false alarm rate). For the analysis of the stimulation effects, the *d′* scores in the noTMS condition were used as baseline and subtracted from the *d′* scores in the corresponding TMS conditions. Additionally, mean reaction times were analyzed to ensure that speed-accuracy trade-offs did not contribute to any differences in accuracy as assessed by *d′*. For reaction times, only trials with correct responses were included. Measures were computed separately for the different TMS conditions, retrocue types, and for cued and neutral trials. Neutral trials were identical in all blocks of trials, and only differed in that they were interleaved with different types of cued trials. However, neutral trials were analyzed separately for the different TMS conditions and retrocue types.

## Results

### ITI Control Experiment

**Figure 2:**
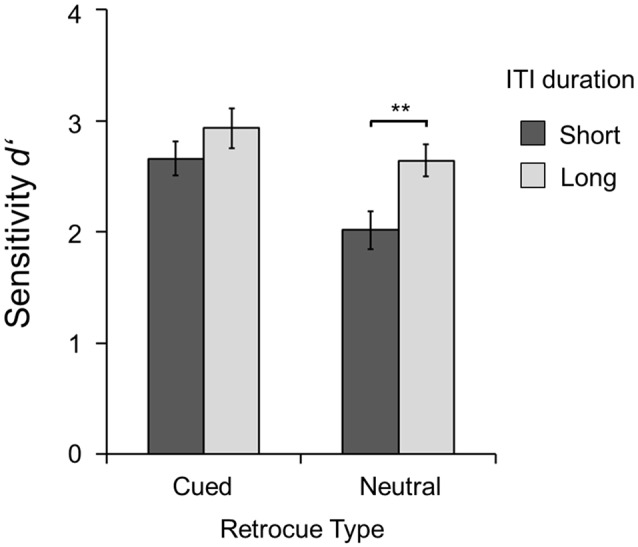
**Results of the inter-trial interval (ITI) control experiment.** Sensitivity of change detection (*d′*) is shown separately for short (dark gray) and long (light gray) ITI durations, and for cued (left) and neutral (right) trials. Error bars show the standard errors of the means. Asterisks mark significant differences between short and long ITIs (**p < 0.01).

Figure 2 shows the sensitivity of change detection (*d′*) for short and long ITIs, separately for cued and neutral trials. A two-way repeated measures analysis of variance (ANOVA) with the factors retrocue type (cued vs. neutral) and ITI duration (short vs. long) showed that performance was better in cued than in neutral trials (*F*_(1,15)_ = 24.84, *p* < 0.001, *η*^2^ = 0.62) and overall it was also better with long ITIs than with short ITIs (*F*_(1,15)_ = 10.72, *p* = 0.005, *η*^2^ = 0.42). An interaction (*F*_(1,15)_ = 4.83, *p* = 0.044, *η*^2^ = 0.24) revealed that the performance with long and short ITIs differed between cued and neutral trials. Follow-up *t*-tests showed that performance was better in cued than in neutral trials with both short ITIs (*t*_(15)_ = 5.66, *p* < 0.001) as well as long ITIs (*t*_(15)_ = 2.22, *p* = 0.022). Importantly, sensitivity (*d′*) was significantly better with long ITIs than with short ITIs (*t*_(15)_ = 4.04, *p* = 0.001) only in neutral trials, whereas it was equivalent with long and short ITIs in cued trials (*t*_(15)_ = 1.69, *p* = 0.111). Thus, ITI duration improved performance in neutral trials, but not in cued trials. Presumably, the long ITI reduced intertrial interference, improving performance when memory load was high (i.e., in neutral trials), but not when memory load was already essentially reduced to one item (i.e., in cued trials). Our statistical analyses of the main experiment were consequently designed in such a way that this differential effect of ITI duration did not affect the conclusions. In particular, the analyses testing for region-specific TMS-induced effects were not performed on the retrocueing benefits (*d′* scores in cued trials − *d′* scores in neutral trials), but separately for cued and neutral trials.

### Main Experiment

**Figure 3:**
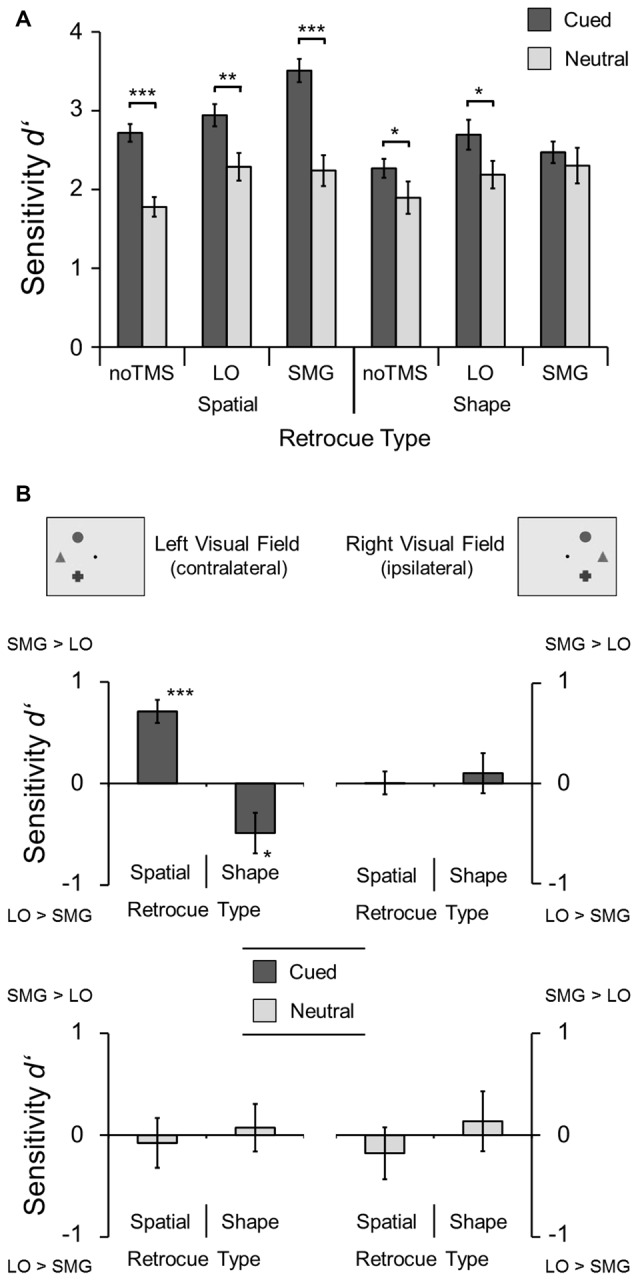
**Results of the main experiment. (A)** Sensitivity of change detection (*d′*) shown for the different retrocue types and TMS conditions. Asterisks mark significant differences between cued and neutral trials (*p < 0.05; **p < 0.01; ***p < 0.001; one-tailed *t*-tests). **(B)** Differential effects of the two TMS sites relative to the no-TMS baseline (SMG minus LO), shown separately for left- and right-hemifield trials and for cued (dark gray, upper row) and for neutral trials (light gray, bottom row). Positive values indicate improved performance with TMS to SMG, negative values indicate improved performance with TMS to LO. Asterisks mark significant differences from zero (two-tailed *t*-tests). Error bars show standard errors of the means.

Figure 3A shows the sensitivity of change detection (*d′*) for the two retrocue types (spatial vs. neutral) and for each TMS condition (noTMS vs. LO vs. SMG), separately for cued and neutral trials. Three analyses were performed on these data. First, to test whether there was a general improvement in performance in cued compared to neutral trials for both types of cues, a two-way repeated measures ANOVA with the factors retrocue information (cued vs. neutral) and retrocue type (spatial vs. shape) was performed on the *d′* scores in the noTMS condition (see Figure 3A). Indeed, *d′* scores were higher in cued than in neutral trials (*F*_(1,10)_ = 25.23, *p* = 0.001, *η*^2^ = 0.72). An interaction revealed that this difference was larger for spatial retrocues (*F*_(1,10)_ = 8.19, *p* = 0.017). Follow-up *t*-tests (one-tailed) confirmed that there were, as expected, significant benefits in the sensitivity of change detection (*d′*) for both shape (*t*_(10)_ = 2.24, *p* = 0.0245) as well as spatial retrocues (*t*_(10)_ = 5.84, *p* < 0.001). The corresponding pattern of results was observed for reaction times. Reaction times were faster in cued than in neutral trials (*F*_(1,10)_ = 64.58, *p* < 0.001, *η*^2^ = 0.87), and this difference was larger for spatial retrocues *F*_(1,10)_ = 7.39, *p* = 0.022, *η*^2^ = 0.43). *T*-tests confirmed that there were significant benefits in terms of reaction time for both shape (*t*_(10)_ = 7.75, *p* < 0.001) and spatial retrocues (*t*_(10)_ = 5.13, *p* < 0.001). Moreover, reaction times were faster in spatial retrocue blocks than in shape retrocue blocks (*F*_(1,10)_ = 23.09, *p* = 0.001, *η*^2^ = 0.70). Thus, participants were able to attentively select a task-relevant item based on either location or shape, yielding improved memory performance for that item.

Second and third, to test for overall effects of the stimulation, two-way repeated measures ANOVAs with the factors retrocue type (spatial vs. shape) and TMS condition (noTMS vs. LO vs. SMG) were computed separately for cued and neutral trials (see “ITI Control Experiment” in Materials and Methods Section and “ITI Control Experiment” in Results Section; see Figure 3A). For neutral trials, there was a significant main effect of TMS condition (*F*_(2,20)_ = 4.76, *p* = 0.02, *η*^2^ = 0.32). Subsequent pairwise comparisons revealed a significant difference between noTMS and LO (−0.40 ± 0.13, *p* = 0.041), and the difference between noTMS and SMG just failed to reach significance (−0.43 ± 0.15, *p* = 0.053). Performance for trials with stimulation of LO and SMG did not differ (−0.03 ± 0.18, *p* = 1). This overall enhancement in TMS blocks compared to noTMS blocks in neutral trials might be due to the longer ITI duration, and not an effect of the stimulation *per se* (see “ITI Control Experiment” in Results Section). There was neither a significant main effect of retrocue type nor an interaction for neutral trials. For cued trials, performance was better with spatial retrocues than with shape retrocues, as shown by a main effect of retrocue type (*F*_(1,10)_ = 55.19, *p* < 0.001, *η*^2^ = 0.85). There was also a main effect of TMS condition, with significant differences between noTMS and LO (−0.33 ± 0.08, *p* = 0.006) and between noTMS and SMG (−0.50 ± 0.10, *p* = 0.001), but not between LO and SMG (−0.17 ± 0.11, *p* = 0.55). Our main interest, however, was in investigating differential TMS-induced effects on attentional selection based on location vs. shape. Indeed, a significant interaction (*F*_(2,20)_ = 6.08, *p* = 0.009, *η*^2^ = 0.38) revealed that the effects of TMS condition differed between retrocue types and more specific analyses were performed to further elucidate this interaction (see below). The same ANOVAs were computed for reaction times. For both cued as well as neutral trials, there were only significant main effects of retrocue type (*cued* *F*_(1,10)_ = 11.58, *p* = 0.007, *η*^2^ = 0.54;* neutral* *F*_(1,10)_ = 5.87, *p* = 0.036, *η*^2^ = 0.37) and there were neither significant effects of TMS condition nor interactions. Thus, there was no speed-accuracy trade-off, and TMS did not affect reaction times.

In order to specifically test for region-specific differential TMS-induced effects while simultaneously controlling for non-specific TMS effects, we subtracted the *d′* values in LO trials from the values in SMG trials after no-TMS baseline correction. This was done separately for the different retrocue types and for the left- and right-hemifield trials (Figure 3B). Note that positive values indicate a greater improvement in performance for TMS over SMG, whereas negative values indicate a greater improvement in performance for TMS over LO. Two-way repeated measures ANOVAs with the factors retrocue type (spatial vs. shape) and visual field (left vs. right) were computed separately for cued trials (Figure 3B, upper row) and for neutral trials (Figure 3B, bottom row). For cued trials, a significant main effect of retrocue type (*F*_(1,10)_ = 10.45, *p* = 0.009, *η*^2^ = 0.51) confirmed that values were higher (and positive) for spatial retrocues, and lower (and negative) for shape retrocues. Moreover, there was a significant interaction of retrocue type and visual field (*F*_(1,10)_ = 20.75, *p* = 0.001, *η*^2^ = 0.68), and follow-up *t*-tests against zero revealed that the site-differentiated enhancement was only observed for the left visual field (contralateral to TMS sites): a positive value for spatial retrocues (*t*_(10)_ = 6.23, *p* < 0.001) indicated relatively enhanced performance with TMS to SMG, and a negative value for shape retrocues (*t*_(10)_ = 2.45, *p* = 0.034) indicated relatively enhanced performance with TMS to LO. No effects were observed for neutral trials.

## Discussion

Our results show that spatial and feature-based attentional selection of VWM representations recruit distinct cortical regions: Stimulation of SMG selectively facilitated spatial attention, whereas stimulation of LO selectively facilitated feature-based attention. This demonstrates, for the first time, that there are specialized cortical modules for the selection of memory contents based on different visual properties.

This cortical dissociation indicates that the basic differentiation between feature-based and spatial attention that has long been established for the perceptual domain also applies to the mnenomic domain. Specifically, SMG and LO have previously been implicated in attentional orienting based on object location and shape in the external world (Chambers et al., 2004; Murray and Wojciulik, 2004; Schenkluhn et al., 2008), which suggests that spatial and feature-based attentional mechanisms utilize similar neural machinery when operating on perceptual input and on mnemonic representations.

From a broader perspective, the idea of overlapping perceptual and VWM attentional systems is consistent with reports of highly overlapping activations for orienting attention in perception and in VWM, involving a large network of frontal, parietal and occipital areas (Lepsien and Nobre, 2006). Our results reveal a specialization of certain brain areas within the neural network involved in attentional selection in VWM with respect to the type of attended stimulus characteristic. This finding can also be seen as being in line with what has been shown for the perceptual domain. Studies typically report the activation of a largely overlapping network, indicating a common control system, with subregions or populations of neurons within this network that are preferential or specific for controlling either spatial or feature-based attention (Vandenberghe et al., 2001; Giesbrecht et al., 2003; Slagter et al., 2007). In light of the correspondence between our results and findings on perceptual attention, it would be a parsimonious hypothesis that the neural implementation of spatial and feature-based attentional selection involves overlapping substrates, specifically SMG and LO.

This need not imply that selective attention for visual perception and VWM share identical circuitry. The brain must also be able to differentiate perceptual input from memory, and be able to selectively deploy attention in these two domains. This selective gating might occur at the level of the microcircuitry and output connections of SMG and LO, as well as in the executive control mechanisms that deploy and gate these modules. An obvious candidate for this function might be prefrontal cortex (e.g., Zanto et al., 2011; Gazzaley and Nobre, 2012; Lee and D’Esposito, 2012; Kuo et al., 2014). Interestingly, differences in the neural substrates of attentional selection in perception and VWM have mostly been observed in frontal areas, with increased frontal involvement for orienting attention in VWM (Nobre et al., 2004; Tanoue et al., 2013). Thus, attention for perception and attention for VWM might share circuitry, while the brain also retains the ability to deploy these forms of attention differentially.

The double dissociation between SMG and LO on attentional orienting based on location and shape was only observed for the visual hemifield that was contralateral to the stimulation sites. This lateralization may be due to the nature of the representations that attention operates on when selecting information in VWM, for which hemispheric lateralization has previously been demonstrated (e.g., Gratton, 1998; Vogel and Machizawa, 2004). While such lateralization is common to the visual system, to our knowledge this is the first time a lateralized effect of TMS on directing attention in VWM has been demonstrated. This finding is consistent with electrophysiological studies reporting lateralized event-related and oscillatory activity following the presentation of retrocues, that is, for selecting representations in VWM (Griffin and Nobre, 2003; Poch et al., 2014; Myers et al., 2015).

Our finding of a TMS-induced enhancement of cognitive performance was rather surprising given that previous studies using a similar protocol and/or stimulating SMG or LO have mostly observed an impairment of performance (e.g., Chambers et al., 2004; Schenkluhn et al., 2008; Romei et al., 2010; Mullin and Steeves, 2011; Bona et al., 2014). The mechanisms of TMS are poorly understood, and whether it results in facilitatory or disruptive effects may depend on a number of stimulation parameters (Luber and Lisanby, 2014). Our triple-pulse rTMS may have modulated oscillatory brain activity in the alpha band. Particularly rTMS, delivered at individual alpha frequency, which on average is 10 Hz and thus equal to our stimulation frequency, has been associated with facilitatory effects on cognitive performance (Klimesch et al., 2003; Luber and Lisanby, 2014). Modulating alpha power using anodal transcranial direct current stimulation has been found to improve performance in a change detection task that involved VWM, presumably due to a change in the attentional state (Hsu et al., 2014). Alpha-band oscillations have recently also been specifically implicated in the attentional selection of VWM representations (Myers et al., 2015). In light of evidence linking alpha-band oscillations to inhibitory mechanisms (Sauseng et al., 2009; Klimesch, 2012), it could be that the facilitation of attentional selection was not mediated by an enhancement of the selected representation (i.e., the cued item) but by facilitated inhibition of the nonselected representations (i.e., the uncued items; see also Tseng et al., 2012).

Regardless of the mechanism, the TMS-induced performance enhancement that we observed could be valuable for the development of TMS-based neurorehabiliation therapies for VWM deficits. TMS-based rehabilitation therapies are still in the early stages of development, but there have been a number of successful uses (Luber and Lisanby, 2014). Establishing the association between a particular TMS protocol and enhancement of a specific cognitive function is an important first step.

When interpreting these findings, we considered several concerns that are not directly related to the TMS-induced effects. First, one might argue that a verbal strategy was adopted, and that participants accordingly memorized the names of the colors. However, several previous studies similarly used categorical colors as the feature to be memorized, and concluded that performance in such tasks relies on VWM (e.g., Ikkai et al., 2010; Kuo et al., 2012; Heuer and Schubö, 2016b), rather than on verbal working memory (e.g., Luck and Vogel, 1997; Luria et al., 2010). Further, it seems more likely that TMS over LO and SMG (which are well-known “highlevel” visual areas) would have affected VWM than a verbal strategy.

Second, the retrocueing benefits in the noTMS condition were considerably smaller for shape retrocues than for spatial retrocues. A potential explanation for this observation is that some participants memorized color-location bindings and ignored shape information. However, out of the 11 participants, only three did not show a benefit with shape retrocues in the noTMS condition, and only one participant did not show a benefit with stimulation of LO. Thus, shape information was clearly available to make use of the shape retrocue, and there was no indication that participants adopted the strategy of ignoring shape information altogether. There is evidence indicating that even task-irrelevant features of objects are automatically encoded, with only subsequent maintenance being under voluntary control (Xu, 2010; Marshall and Bays, 2012). Given that shape was required to make use of the retrocue, it seems reasonable to assume that this feature was encoded and maintained along with color and location in an object-based manner (see also Luck and Vogel, 1997; Luria and Vogel, 2011). In support of this, two recent studies showed that tasks that emphasize feature binding (which was the case here) encourage the storage of integrated objects (Vergauwe and Cowan, 2015; Balaban and Luria, 2016). In a previous study using a very similar design (Heuer and Schubö, 2016a), we observed equivalent overall benefits for shape and spatial retrocues. We did not systematically analyze individual differences in that study, but it does seem that some people preferred one type of retrocue over the other, yielding larger benefits for this preferred cue type.

To conclude, we have shown that different cortical areas subserve spatial and feature-based selection of VWM representations, indicating that these are distinct attentional mechanisms. The correspondence between our findings and what has been established for perceptual attention suggests that these types of top-down control over mnemonic representations and perceptual input are similarly implemented in parietal and occipital cortex. In general, these results provide novel insight into how attentional mechanisms operating on different kinds of information optimize the visual system, allowing for an efficient use of the limited VWM system.

## Author Contributions

AH, AS and JDC designed research, AH collected and analyzed data, AH, AS and JDC wrote the article.

## Conflict of Interest Statement

The authors declare that the research was conducted in the absence of any commercial or financial relationships that could be construed as a potential conflict of interest.
